# Enhanced alcohol degradation and hepatic protective effects of an *Acetobacter Pasteurianus*-derived product, CureZyme-ACE, in an acute intoxication rat model

**DOI:** 10.1186/s42826-020-00050-4

**Published:** 2020-06-05

**Authors:** Eun-Jong Jeon, Yun-Sang Cho, Ae-Hyang Kim, Jae-Min Shim, Yi-Soo Kim, Zhe Piao, Young Chul Shin, Jungkee Kwon

**Affiliations:** 1 Department of Laboratory Animal Medicine, College of Veterinary Medicine, Jeonbuk National UniversityJeonbuk, 54596 Iksan, Republic of Korea; 2 Amicogen, Inc., Jinsung, Jinju, Republic of Korea

**Keywords:** Alcohol, Acetaldehyde, Intoxication, *Acetobacter Pasteurianus*, CureZyme-ACE

## Abstract

Excessive alcohol consumption induces acute intoxication and various hepatic diseases. In this study, we investigated the effect of the CureZyme-ACE (CA), *Acetobacter Pasteurianus* (AP)-derived product, in acute intoxication rats. The ethanol and acetaldehyde levels of serum were lower in rats treated with CA than those who only treated ethanol. The activities of alcohol dehydrogenase and acetaldehyde dehydrogenase also recovered faster in the CA group than only-ethanol group. The transaminase levels (AST, ALT) in the CA group were significantly lower than only-ethanol group. In addition, Hepatic histological analyses and stomach wall were demonstrated that the CA-treated group recovered faster than only-ethanol group. With regard to most characteristics, we found that CA had dose-dependent effects. At high concentrations of CA, there were no differences in the tested parameters compared to those of normal rats. These findings indicate that CA reduces the serum alcohol concentration and some of the hepatic damage caused by alcohol intoxication.

## Introduction

Alcohol has long been a favorite beverage across the world. Drinking in moderation can actually help with blood circulation. However, large doses of alcohol at one time can cause acute intoxication, acute alcoholic hangover, nausea, vomiting, dizziness, headache, and muscle aches [1]. Alcohol is mainly absorbed in the digestive tract, with up to 30% absorbed in the upper gastrointestinal tract and 60% in the small intestine. This absorbed alcohol is metabolized by the liver. The remaining 10% is excreted through one’s breath, urine, and sweat. Absorbed alcohol is oxidized to acetaldehyde by alcohol dehydrogenase (ADH). Acetaldehyde is then oxidized to acetic acid by acetaldehyde dehydrogenase (ALDH). Acetaldehyde can be used as an energy source in the body [2]. However, certain allelic variations in the ALDH2 gene can result in very low enzyme activity, which ultimately leads to acetaldehyde accumulation [3]. Acetaldehyde and nicotinamide adenine dinucleotide (NADH) are produced in the process of alcohol oxidization and are both toxic to the body. These molecules damage hepatocytes and lead to reactive oxygen species (ROS) production and hangover [4]. Excess alcohol consumption also damages the mucous membrane of the digestive tract, resulting in malnutrition. As a result, alcohol abuse has toxic effects on all organs and tissues and causes many diseases, including liver necrosis, pancreatitis, and cardiomyopathy. In addition, alcohol-induced damage to the liver disturbs various metabolic pathways and generally destroys the body’s metabolic balance [5]. Given these data, there is growing interest to identify natural products that can reduce alcoholism or hangover. Therefore, recent researchers have investigated natural products that rapidly degrade ethanol and acetaldehyde by simultaneously activating ADH and ALDH [6, 7]. However, in most cases, there is accumulation of toxic acetaldehyde when ADH is more highly activated than ALDH. Therefore, none of the tested substances has produced remarkable effects thus far.

In Korea, *Acetobacter Pasteurianus* (AP) is the only generally recognized as a safe (GRAS) strain for use in vinegar by the Ministry of Food and Drug Safety (MFDS) [8]. *Acetobacter Pasteurianus* (AP) is the most important acetic acid bacteria in the vinegar industry. It has also been reported to have high alcohol tolerance and strong resistance to acetic acid. Acetic acid is produced using ADH, NAD+, and ALDH [9]. Therefore, in this study, we investigated the effect of the *Acetobacter Pasteurianus*-derived product, CureZyme-ACE (CA) in rats with acute alcohol intoxication.

## Materials and methods

### CA development

**Fig. 1:**
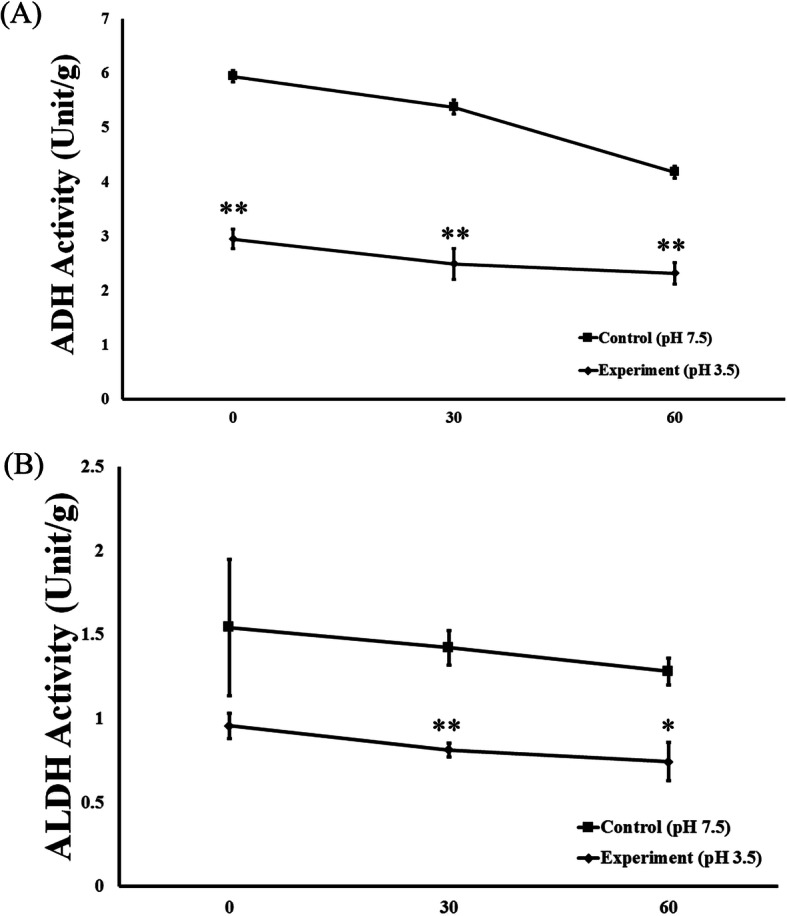
Stability of CureZyme-ACE (CA) in an acidic environment. (**a)** ADH activity, (**b**) ALDH activity. Control: pH 7.5 environment, Experiment: pH 3.5 environment. Data were expressed as mean ± SE (*n* = 3). ^*^p < 0.05, ^**^p < 0.01, relative to Control

AP vinegar amicogen line 2 (VA2) was supplied from Amicogen, Inc. A single colony was obtained from YPM agar plate (Yeast extract 0.5%, Peptone 0.3%, Mannitol 2.5%, Agar 1.5%) with reactivated lyophilized powder. To conduct acetic acid fermentation, *Acetobacter Pasteurianus* (AP) were inoculated into the YPM broth at 2% and cultured at 26 °C with shaking at 200 rpm for 18 h. The culture broth was shake-cultured in YSGM broth (Yeast extract 0.5%, Soytone 1%, D-glucose 5%, Manganese sulfate 0.01%). The cell pellet was obtained by centrifuging the culture broth (10,000 xg, 20 min, 4 °C) and was suspended in 1 mM potassium phosphate buffer (pH 7.4). The suspension was then homogenized (MN20A, Picomax, Seoul, Korea) at 4 °C under 15,000 psi. Maltodextrin was added to the suspension and mixed for 10 min. This solution was then freeze-dried to produce AP powder, which was used in the experiment (Product name: CureZyme-ACE, CA). To confirm CA stability in acidic conditions, CA was reacted in 3 M Tris-HCl (pH 3.5) at 36 °C, 150 rpm for 0, 30, and 60 min. The determination of ADH and ALDH activity of CA was carried out according to the Enzymatic Assay of Alcohol Dehydrogenase (ADH) and Enzymatic Assay of Aldehyde Dehydrogenase (ALDH) provided by Sigma Aldrich (Fig. 1).

### Animals and treatment

Six-week-old male Sprague-Dawley (SD) rats were purchased from Damul Science (Daejeon, Korea). All of the animals had free access to standard laboratory pellet and water. The animals were housed under specific pathogen-free conditions on a 12 h light/dark cycle at constant temperature (25 ± 2 °C) and humidity (about 60%). All of the animals were cared for in accordance with institutional ethical guidelines for the care and use of experimental animals at Jeonbuk National University. The rats were acclimatized for the first week. Commercial product Dawn808 (Glami co., Gangwon, Korea) [10] was used as a positive control [11]. The animals were randomly divided into the following five groups: Normal, Control (ethanol 4 g/kg, oral administration), L-CA (ethanol 4 g/kg + CA 103 mg/kg, oral administration), H-CA (ethanol 4 g/kg + CA 309 mg/kg, oral administration), PC (Positive control, ethanol 4 g/kg + Dawn808 12 ml/kg, oral administration). Rats were treated with CA and Dawn808 at 30 min before ethanol administration. The blood samples were collected from the tail vein of normal and acutely intoxicated rats after 0, 0.5, 1, 3, 5, and 8 h of ethanol treatment. The serum samples were immediately separated from the blood by centrifugation. Rat livers were perfused with saline and collected after 1 and 8 h, placed in liquid nitrogen and stored at − 72 °C until analyses.

### The concentrations of ethanol and acetaldehyde

The serum concentrations of ethanol and acetaldehyde were measured with the commercial assay kit using the EnzyChrom™ Ethanol Assay Kit and EnzyChrom™ Acetaldehyde Assay Kit (BioAssay System, CA, USA) according to the manufacturer’s instructions. The absorbance of each well was measured at 565 nm using a microplate reader. The area under the serum concentration-vs-time curve (AUC) of the serum ethanol concentration-time was calculated with reference to the previous paper [12].

### The activities of ADH and ALDH

The activities of ADH and ALDH in the serum were determined using ADH and ALDH activity assay kits (Abcam, Cambridge, MA) according to the manufacturer’s instructions. The absorbance of each well at 450 nm was measured with a microplate reader. The AUC of the serum ethanol concentration-time was calculated with reference to the previous paper [12].

### Aspartate transaminase (AST) and alanine aminotransferase (ALT)

The serum levels of AST and ALT are indicators of liver function that were measured using a serum AST and ALT kit (ASAN Pharmaceutical Co., Ltd., Seoul, Korea) according to the manufacturer’s instructions. The absorbance of each well at 490 nm was measured using a microplate reader.

### Hepatic tissue histology

The hepatic tissues were sampled after 1 and 8 h of ethanol exposure. These tissues were fixed in 10% buffered formalin. All of the tissues were embedded in paraffin and cut at 4–5 μm. The sections were deparaffinized using a xylene and graduated alcohol series to water. The sections were stained with hematoxylin and eosin (H & E) and evaluated using a standard light microscope.

### Statistical analysis

The Normal and Control groups were compared, and the Control and CA groups were compared separately. Statistical analyses were performed using Student’s t-test and repeated-measures ANOVA followed by a Bonferroni test. The data are expressed as mean ± SEM. *P* values < 0.05 were considered statistically significant [13].

## Results

### Alcohol metabolism

**Fig. 2:**
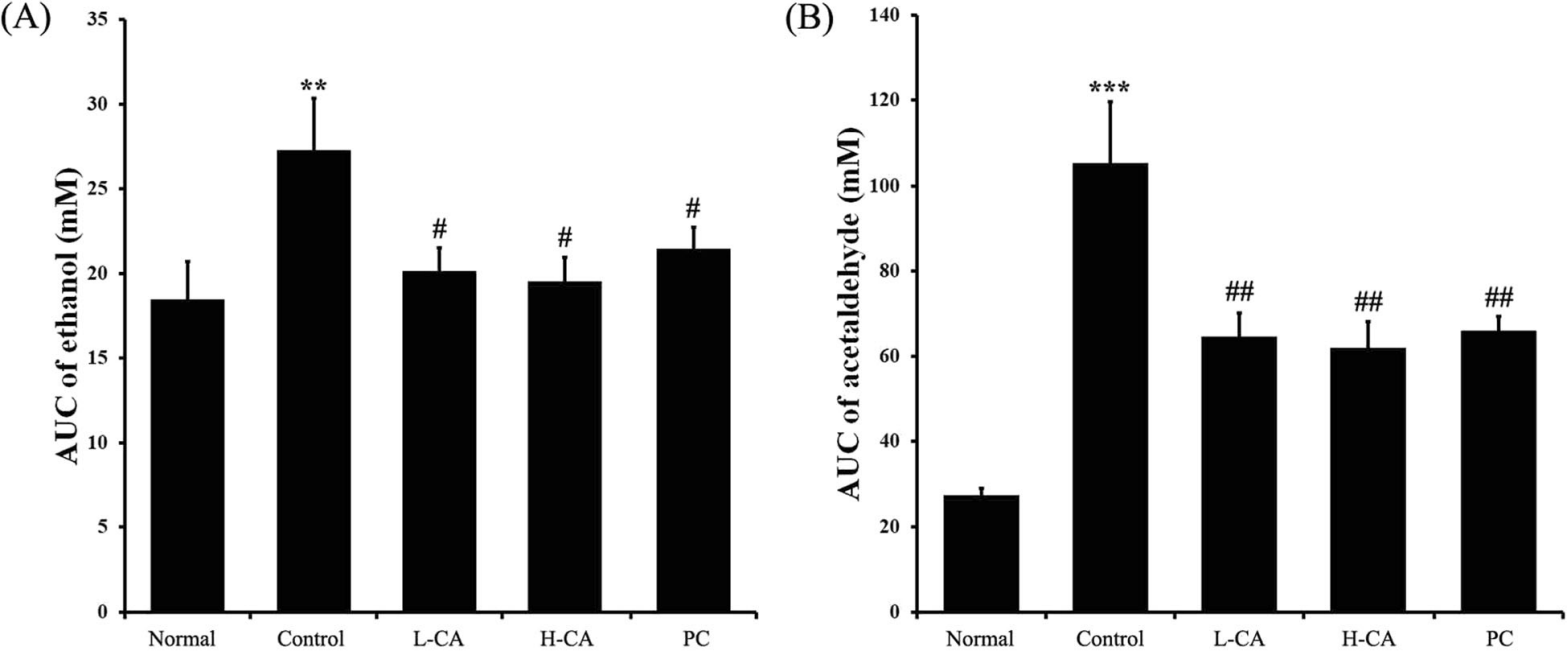
Area under the serum concentration vs. time curves (AUC) of CureZyme-ACE (CA). (**a**) AUC of ethanol, (**b**) AUC of acetaldehyde. Normal: Saline, Control: Ethanol, L-CA: Low concentration CA + ethanol, H-CA: High concentration CA + ethanol, PC: Dawn808 + ethanol. Data were expressed as mean ± SE (*n* = 10). ^**^p < 0.01, ^***^p < 0.001, relative to Normal. ^#^*p* < 0.05, ^##^*p* < 0.01, relative to Control

To investigate the effect of CA on alcohol metabolism, we measured the serum ethanol and acetaldehyde levels. As expected, the serum ethanol concentration increased rapidly after ethanol administration and decreased with time in all of the groups except the Normal group (Table 1). The serum ethanol level increased rapidly 30 min after administration in the CA groups and PC group. However, this level was lower than in the Control group. The L-CA group was similar to the PC group. The H-CA group showed a significantly lower tendency. After 1 h, the ethanol level of the CA groups decreased in a dose-dependent manner. This decrease was significantly greater in the H-CA group than it was in the PC group. The AUC of serum ethanol-time was significantly decreased in the CA and PC groups compared to that of the Control (Fig. 2a). Acetaldehyde concentration also increased rapidly after ethanol administration in all groups except the Normal group and decreased with time (Table 2). The increase in acetaldehyde level was significantly lower in the CA and PC groups than it was in the Controls. Five hours after ethanol administration, the H-CA showed lower tendencies than did the PC. The AUC of serum acetaldehyde-time was significantly decreased in the CA and PC groups compared to the Controls (Fig. 2b).

**Table 1:** Effect of CureZyme-ACE (CA) on serum ethanol. Normal: Saline, Control: Ethanol, L-CA: Low concentration CA + ethanol, H-CA: High concentration CA + ethanol, PC: Dawn808 + ethanol. *p < 0.05, ***p < 0.001, relative to Normal. #*p* < 0.05, ##*p* < 0.01, ###*p* < 0.001, relative to Control. $*p* < 0.05, $$*p* < 0.01, relative to PC

			Time(h)			
	**0**	**0.5**	**1**	**3**	**5**	**8**
**Normal**	2.50 ± 0.17	2.36 ± 0.12	2.44 ± 0.13	2.43 ± 0.38	2.42 ± 0.28	2.40 ± 0.15
**Control**	2.45 ± 0.16	8.07 ± 0.64^***^	4.44 ± 0.27^***^	3.74 ± 0.14^*^	3.55 ± 0.43^*^	3.47 ± 0.35^*^
**L-CA**	2.35 ± 0.16	4.67 ± 0.44^###^	2.72 ± 0.35^##^	2.69 ± 0.23^#^	2.42 ± 0.16^#^	2.61 ± 0.34
**H-CA**	2.37 ± 0.14	3.47 ± 0.44^###$^	2.39 ± 0.20^##$$^	2.47 ± 0.18^##^	2.43 ± 0.27^#^	2.47 ± 0.46^#^
**PC**	2.39 ± 0.22	4.88 ± 0.58^##^	3.40 ± 0.44^#^	2.50 ± 0.35^#^	2.56 ± 0.30^#^	2.46 ± 0.20^#^

**Table 2:** Effect of CureZyme-ACE (CA) on serum acetaldehyde. Normal: Saline, Control: Ethanol, L-CA: Low concentration CA + ethanol, H-CA: High concentration CA + ethanol, PC: Dawn808 + ethanol. ***p < 0.001, relative to Normal. #*p* < 0.05, ##*p* < 0.01, ###*p* < 0.001, relative to Control. $*p* < 0.05, relative to PC

Time(h)						
	0	0.5	1	3	5	8
**Normal**	3.46 ± 0.20	3.40 ± 0.36	3.36 ± 0.53	3.39 ± 0.36	3.36 ± 0.22	3.46 ± 0.29
**Control**	3.46 ± 0.17	14.03 ± 1.97^***^	17.23 ± 2.28^***^	14.09 ± 3.34^***^	13.60 ± 2.70^***^	10.36 ± 3.10^***^
**L-CA**	3.43 ± 0.33	9.13 ± 0.84^##^	11.42 ± 2.81^##$^	9.00 ± 1.75	8.62 ± 0.42^#^	4.21 ± 0.91^##$^
**H-CA**	3.61 ± 0.39	7.98 ± 1.55^##^	12.97 ± 1.61^#$^	8.91 ± 0.57^#^	6.64 ± 1.79^##^	4.25 ± 1.28^##$^
**PC**	3.79 ± 0.69	9.60 ± 0.46^##^	13.45 ± 2.05^#^	9.09 ± 0.59^#^	8.43 ± 1.82	2.78 ± 0.32^###^

### ADH and ALDH activities

We measured the serum activities of ADH and ALDH in order to determine the effect of CA on the activity of the alcohol degradation enzyme. The CA groups had significantly higher ADH activity than did the Control group at 0, 0.5, 1, 3, 5, and 8 h after ethanol treatment (Table 3). In contrast, the serum ALDH activities of the CA groups were significantly lower than were those in the Control group (Table 4). Over the course of 8 h, the CA groups recovered to Normal levels. In this study, the ADH and ALDH activities recovered rapidly in the CA group after alcohol administration.

**Table 3:** Serum ADH activity with CureZyme-ACE (CA) use. Normal: Saline, Control: Ethanol, L-CA: Low concentration CA + ethanol, H-CA: High concentration CA + ethanol, PC: Dawn808 + ethanol. ^**^p < 0.01, ^***^p < 0.001, relative to Normal. ^#^*p* < 0.05, ^##^*p* < 0.01, ^###^*p* < 0.001, relative to Control

			Time (h)			
	**0**	**0.5**	**1**	**3**	**5**	**8**
**Normal**	19.23 ± 3.49	22.39 ± 5.72	20.75 ± 1.50	19.75 ± 3.46	21.60 ± 2.97	20.16 ± 2.76
**Control**	18.45 ± 3.41	20.21 ± 8.73	11.09 ± 3.01^**^	8.97 ± 1.73^***^	13.95 ± 2.09^**^	18.38 ± 1.87
**L-CA**	18.54 ± 3.24	32.84 ± 2.26	32.75 ± 7.51^###^	14.87 ± 1.79^#^	23.41 ± 3.123^##^	21.88 ± 7.55
**H-CA**	20.54 ± 3.43	28.26 ± 2.78	24.97 ± 3.62^###^	16.90 ± 3.32^#^	27.16 ± 2.25^###^	23.74 ± 3.47
**PC**	17.73 ± 5.84	35.18 ± 2.64^#^	26.98 ± 3.53^###^	15.41 ± 5.75	23.50 ± 1.70^##^	21.06 ± 2.20

**Table 4:** Serum ALDH activity with CureZyme-ACE (CA) use. Normal: Saline, Control: Ethanol, L-CA: Low concentration CA + ethanol, H-CA: High concentration CA + ethanol, PC: Dawn808 + ethanol. ^*^p < 0.05, ^**^p < 0.01, ^***^p < 0.001, relative to Normal. ^#^*p* < 0.05, ^##^*p* < 0.01, ^###^*p* < 0.001, relative to Control

			Time (h)			
	**0**	**0.5**	**1**	**3**	**5**	**8**
**Normal**	14.52 ± 2.74	14.09 ± 2.10	13.50 ± 1.22	13.80 ± 1.37	13.89 ± 2.62	13.96 ± 1.68
**Control**	14.97 ± 1.55	2.85 ± 1.49^***^	4.23 ± 1.44^***^	2.89 ± 0.96^***^	7.48 ± 0.08^**^	8.16 ± 0.91^*^
**L-CA**	14.37 ± 0.59	16.40 ± 0.76^###^	10.51 ± 1.75^#^	6.51 ± 2.29^#^	11.44 ± 3.85	14.06 ± 2.52^#^
**H-CA**	14.14 ± 1.11	16.43 ± 0.97^###^	8.98 ± 1.73^#^	7.06 ± 1.55^#^	11.54 ± 2.05^#^	14.39 ± 4.99
**PC**	13.41 ± 0.57	15.57 ± 1.70^###^	7.44 ± 3.07	6.15 ± 2.46	9.65 ± 0.77^#^	14.30 ± 1.37^#^

### Evaluation of hepatic damage

**Fig. 3:**
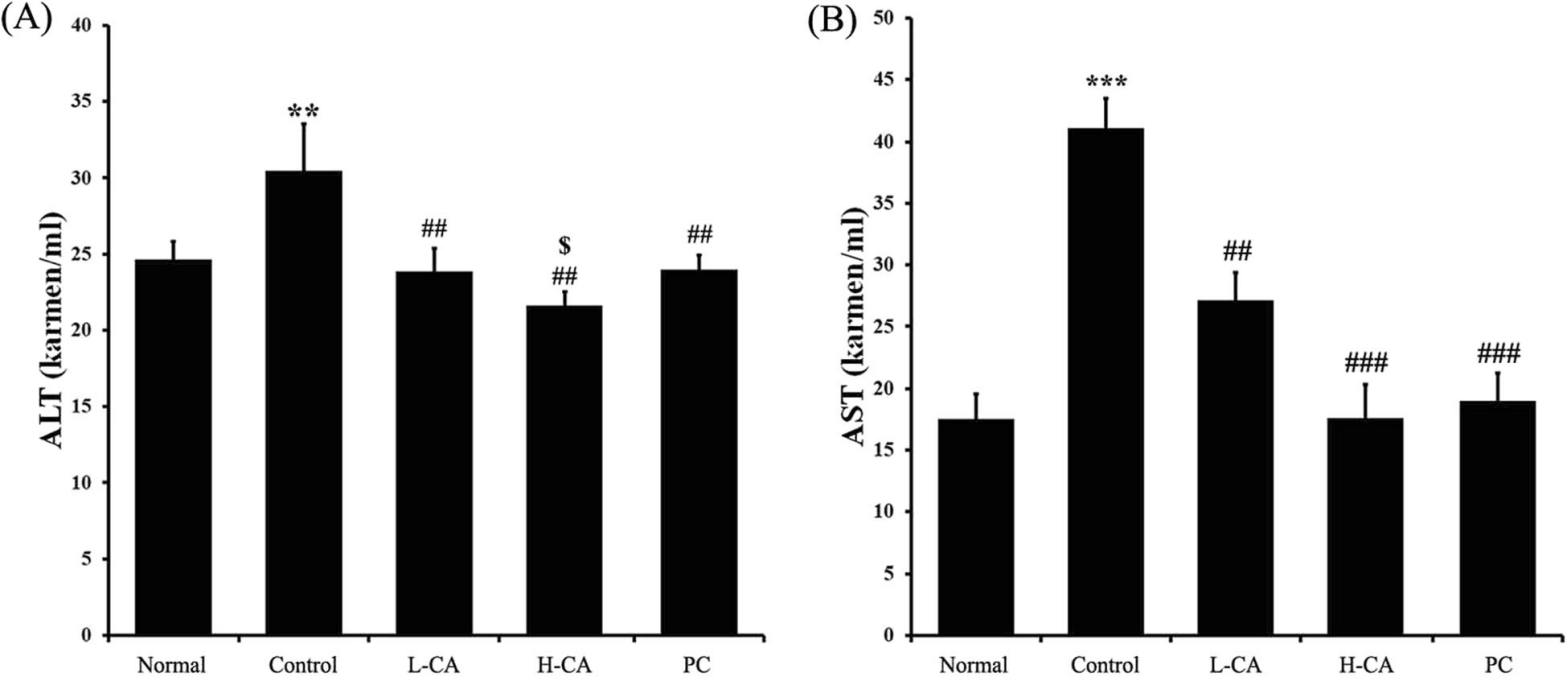
Effects of CureZyme-ACE (CA) on serum. (**a**) ALT, (**b**) AST. Normal: Saline, Control: Ethanol, L-CA: Low concentration CA + ethanol, H-CA: High concentration CA + ethanol, PC: Dawn808 + ethanol. Data were expressed as mean ± SE (*n* = 10). ^**^p < 0.01, ^***^p < 0.001, relative to Normal. ^##^*p* < 0.01, ^###^*p* < 0.001, relative to Control. ^$^*p* < 0.05, relative to PC

We measured the serum levels of AST and ALT to determine the degree of liver damage. These serum transaminase levels were significantly lower in the CA groups than they were in the Control group (Fig. 3). The H-CA group had a significantly lower ALT concentration than that of the PC (Fig. 3a).

### Histological examination of liver and stomach wall change

**Fig. 4:**
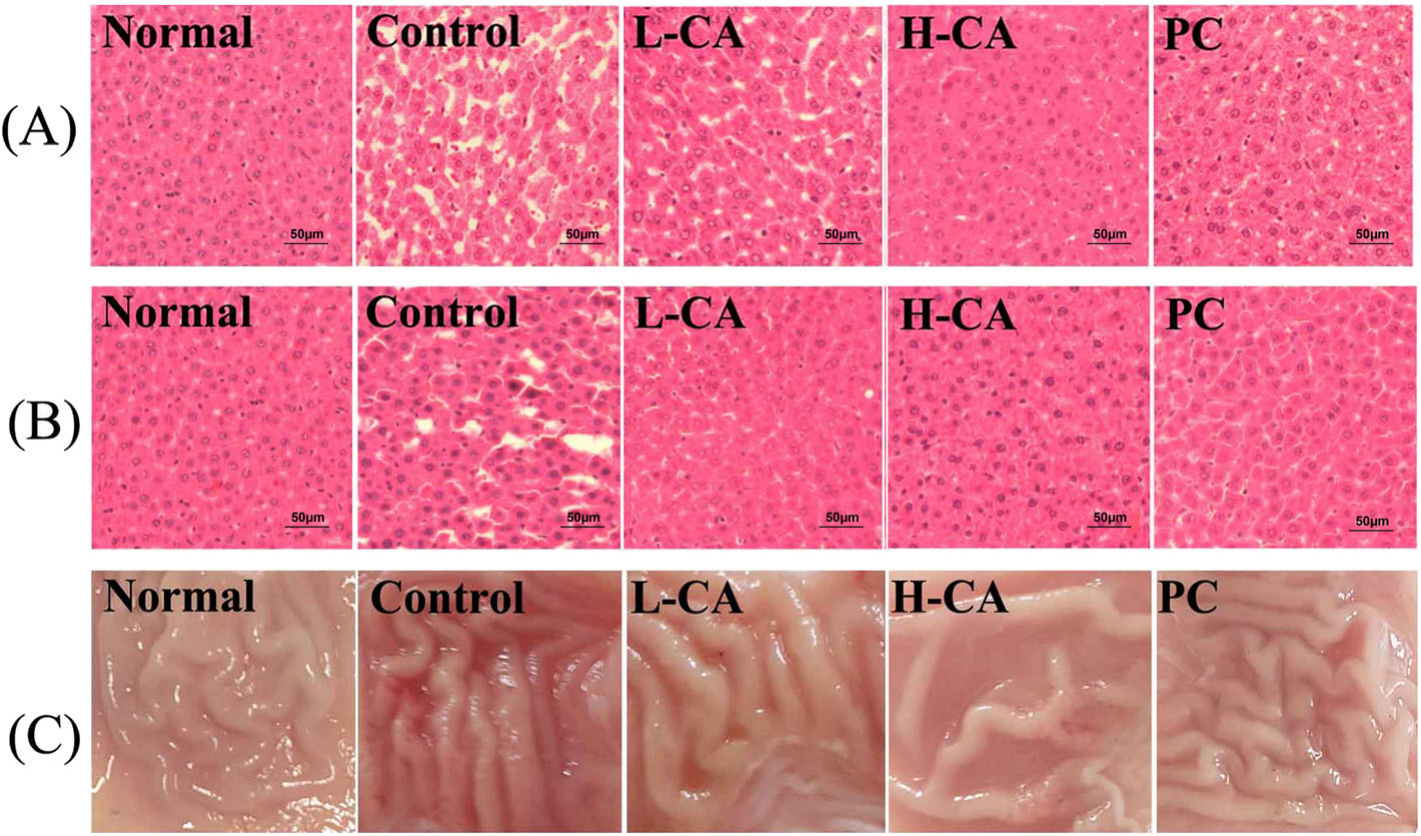
Histological examination of liver and changes in stomach wall after ethanol administration. (**a**) liver histology at 1 h, (**b**) liver histology at 8 h, (**c**) stomach wall change at 8 h. H&E: Hematoxylin and eosin staining. Normal: Saline, Control: Ethanol, L-CA: Low concentration CA + ethanol, H-CA: High concentration CA + ethanol, PC: Dawn808 + ethanol

We performed histological analyses of the liver and changes in the stomach wall to identify alcoholic damages caused by acute alcohol intoxication. In the Control group, there was liver damage, as characterized by prominent microvesicular steatosis with necrosis (Fig. 4a, b). In contrast, only limited microvesicular steatosis was observed in the CA group at 1 h after ethanol administration (Fig. 4a). The liver tissues of the CA and PC groups recovered almost completely after 8 h (Fig. 4b). In particular, the histological findings of the H-CA group at 1 h were similar to those of the Normal group (Fig. 4a, b). In the stomach wall, the Control group demonstrated more redness than the other groups. The results of the H-CA group were similar to those of the normal group (Fig. 4c).

## Discussion

Hangover occurs when there is ethanol remaining in the body and causes systemic fatigue, lethargy, thirst, headache, muscle aches and pain, and gastrointestinal symptoms such as nausea and vomiting. Alcohol consumption can change one’s sleep time and biorhythm, ultimately reducing concentration and contributing to depression, anxiety, and irritability [14]. It also can stimulate the sympathetic nervous system and cause seizures, sweating, tachycardia, and elevated systolic blood pressure [15]. The purpose of this study was to investigate the effects of CA in reducing hangover after alcohol consumption. In studies of pharmacokinetic behavior, AUC is an index used to analyze drug absorption, distribution, and metabolism in the body [16]. In this study, the serum ethanol and acetaldehyde concentration decreased against ethanol-induced damage (Tables 1, 2). The AUC of ethanol and acetaldehyde decreased against ethanol induced damage (Fig. 2). These results suggest that CA can reduce or even eliminate hangover symptoms by shortening the residence time of ethanol in the body.

Alcohol metabolism in the liver is controlled by the activity of ADH and ALDH [17]. Excessive alcohol consumption increases the ethanol oxidation and increases the production of oxygen radicals, such as superoxide and hydroxyl radicals, by 4–8 times [18]. These oxygen radicals increase oxidative cell damage, hepatic damage, and cell death, which ultimately results in DNA mutations, carcinogenesis, senescence, arteriosclerosis, and inflammation [19, 20]. Acetaldehyde-induced hepatic damage also lowers ALDH activity. Therefore, there is a vicious cycle in which acetaldehyde is perpetuated [21]. In this study, the ADH and ALDH activities of the CA groups recovered against ethanol-induced damage (Tables. 3, 4). These results suggest that CA treatment allows for rapid degradation of ethanol and acetaldehyde. The increased activity of alcohol metabolic enzymes is expected to maintain low serum ethanol and acetaldehyde concentrations, thereby inhibiting alcohol’s side effects. ALT and AST are two of the main enzymes present in hepatocytes. AST is released into the serum when liver tissue is damaged [22]. ALT release is induced by hepatitis, hepatic necrosis, and cirrhosis [18]. Therefore, AST and ALT are often used as markers of hepatic damage. Alcohol administration, in particular, is reported to increase the serum levels of AST and ALT [20]. In this study, the serum levels of AST and ALT of the CA groups significantly decreased against ethanol-induced damage (Fig. 3). These results suggest that CA not only reduces hangover symptoms by increasing the rate of alcohol metabolism, but also has hepatoprotective properties. Alcohol metabolism leads to overproduction of toxic metabolites, including acetaldehyde, ROS, and NADH. These molecules destroy the mucosal barrier in the gastrointestinal tract, which can deplete the gastric wall mucus [23]. These effects contribute to the progression of alcoholic fatty liver, hepatitis, liver cirrhosis, and liver cancer, possibly resulting in death [24, 25]. In this study, the liver tissues of the CA groups recovered against ethanol-induced damage (Fig. 4). These results demonstrate that CA has protective effects against ethanol-induced damage to the stomach walls and liver by increasing the rate of alcohol metabolism. Based on research finding, it is worth studying that the estimated effect of CA could support human alcoholic liver disease in a larger cohort.

## Conclusions

In this study, we investigated the effects of CA on alcohol degradation activity and alcoholic hepatic damage. CA treatment decreased the effects of ethanol and acetaldehyde. Exposure to CA also led to rapid recovery of the enzyme activity related to alcohol metabolism. Furthermore, CA reduced the degree of transaminase elevation associated with alcohol. The gastric and hepatic histological analyses were consistent with previous results [26–28]. Overall, our findings suggest that CA protects against alcohol’s toxic effects on the stomach and liver, as well as the symptoms of hangover. We believe that CA is therefore more competitive than the commercial product for use in this setting.

## Data Availability

The data that support the findings of this study are available on request from the corresponding author reasonable request.

## Abbreviations

ADH: Alcohol dehydrogenase
ALDH: Acetaldehyde dehydrogenase
ALT: Alanine aminotransferase
AP: Acetobacter pasteurianus
AST: Aspartate transaminase
CA: CureZyme-ACE

## References

[1] Kim KM, Jung HJ, Sung HM, Wee J-H, Kim TY, Kim KM (2014). Study of the antioxidant and alcohol-degrading enzyme activities of soybean sprout sugar solutions. Korean J Food Sci Technol.

[2] Sung HM, Jung HJ, Yun SK, Kim TY, Kim KM, Wee J-H (2014). Effect of a soy-sprout beverage prepared with high-concentrated oxygen water on alcohol metabolism in rats. Korean J Food Sci Technol.

[3] Foroud T, Edenberg HJ, Crabbe JC (2010). Genetic Research: Who Is At Risk for Alcoholism. Alcohol Res Health.

[4] Gemma S, Vichi S, Testai E (2006). Individual susceptibility and alcohol effects: biochemical and genetic aspects. Ann Ist Super Sanità.

[5] Choi G-H, Kim J-G, Kwon S-T (2011). Protective effects of food including Hovenia dulcis on acute alcohol intoxication. J Korean Soc Food Sci Nutr.

[6] Xu BJ, Zheng YN, Sung CK (2005). Natural medicines for alcoholism treatment: a review. Drug Alcohol Rev.

[7] Yoo GJ, Kim SY, Choi A, Son MH, Kim DC, Chae HJ (2009). Effect of *Rhus verniciflua* stokes extract on the alcohol-metabolizing enzyme activities. Korean Soc Biotech Bioeng.

[8] Yim EJ, Jo SW, Lee ES, Park HS, Ryu MS, Uhm TB (2015). Fermentation characteristics of mulberry (*Cudrania tricuspidata*) fruit vinegar produced by acetic acid bacteria isolated from traditional fermented foods. 1 Korean J Food Preserv.

[9] Wang B, Shao Y, Chen T, Chen W, Chen F (2015). Global insights into acetic acid resistance mechanisms and genetic stability of Acetobacter pasteurianus strains by comparative genomics. Sci Rep.

[10] Seo JY, Kim SS, Kim J-S (2014). Enhancement of alcohol metabolism by sprouted peanut extract in SD rats. Prev Nutr Food Sci.

[11] An S-W, Kim Y-G, Kim M-H, Lee B-I, Lee S-H, Kwon H-I (1999). Comparison of hepatic detoxification activity and reducing serum alcohol concentration of Hovenia dulsis $ T_ {HUNB} $ and Alnus japonica Steud. Korean J Med Crop Sci.

[12] Verdière KJ, Roy RN, Dehais F. Detecting pilot's engagement using fnirs connectivity features in an automated vs. manual landing scenario. Front Hum Neurosci. 2018;12. 10.3389/fnhum.2018.00006. 10.3389/fnhum.2018.00006 PMC5788966 29422841

[13] Andrikopoulos S, Blair AR, Deluca N, Fam BC, Proietto J (2008). Evaluating the glucose tolerance test in mice. Am J Physiol Endocrinol Metab.

[14] van Schrojenstein LM, Roth T, Roehrs T, Verster JC (2017). Alcohol hangover, sleep quality, and daytime sleepiness. Sleep Vigilance.

[15] Ml M, Al S, Js T. Alcohol hangover and risk of drinking problems and alcohol use disorder: a systematic review. J Alcohol Drug Depend. 2017;05(01). 10.4172/2329-6488.1000255.

[16] Casey Laizure S, Herring V, Hu Z, Witbrodt K, Parker RB (2013). The role of human carboxylesterases in drug metabolism: have we overlooked their importance. Pharmacotherapy.

[17] Konno S, Chu K, Feuer N, Phillips J, Choudhury M (2015). Potent anticancer effects of bioactive mushroom extracts (phellinus linteus) on a variety of human cancer cells. J Clin Med Res.

[18] Lieber CS (1991). Hepatic, metabolic and toxic effects of ethanol. Alcohol Clin Exp Res.

[19] Choi JS, Yoon TJ, Kang KR, Lee KH, Kim WH, Suh YH (2006). Glycoprotein isolated from Acanthopanax senticosus protects against hepatotoxicity induced by acute and chronic alcohol treatment. Biol Pharm Bull.

[20] Kundu R, Dasgupta S, Biswas A, Bhattacharya A, Pal BC, Bandyopadhyay D (2008). Cajanus cajan Linn. (Leguminosae) prevents alcohol-induced rat liver damage and augments cytoprotective function. J Ethnopharmacol.

[21] Hwang JY, Ham JW, Nam SH (2004). Effect of Maesil (Prunus mume) juice on the alcohol metabolizing enzyme activities. Korean J Food Sci Technol.

[22] Kim SM, Kang SH, Ma JY, Kim JH (2005). A study on the extraction and efficacy of bioactive compound from Hovenia dulcis. Korean J Biotechnol Bioeng.

[23] Bagchi D, Carryl OR, Tran MX, Krohn RL, Bagchi DJ, Garg A (1998). Stress, diet and alcohol-induced oxidative gastrointestinal mucosal injury in rats and protection by bismuth subsalicylate. J Appl Toxicol.

[24] Keshavarzían A, Fields JZ, Vaeth J, Holmes EW (1994). The differing effects of acute and chronic alcohol on gastric and intestinal permeability. Amer J Gastroenterol.

[25] IH K (2008). Pathophysiology of alcoholic liver disease. Clin Mole Hepatol.

[26] Canesso MCC, Lacerda N, Ferreira C, Gonçalves J, Almeida D, Gamba C (2014). Comparing the effects of acute alcohol consumption in germ-free and conventional mice: the role of the gut microbiota. BMC Microbiol.

[27] Choi YJ, Kim N, Lee JY, Nam RH, Seo JH, Lee S (2016). Gastroprotective effects of PMK-S005 against ethanol-induced acute gastric damage in rats. Gut and liver.

[28] Kim E-H, Chung J (2016). Protective effects of Cirsium setidens ethanolic extracts against alcoholic fatty liver injury in rats. Journal of Nutrition and Health.

